# A Novel Mixed Stimulation Pattern for Balanced Pulmonary EIT Imaging Performance

**DOI:** 10.3390/bioengineering13010072

**Published:** 2026-01-08

**Authors:** Zhibo Zhao, Zhijun Gao, Heyao Zhu, Zhanqi Zhao, Meng Dai, Zilong Liu, Feng Fu, Lin Yang

**Affiliations:** 1 Shaanxi Provincial Key Laboratory of Bioelectromagnetic Detection and Intelligent Perception, Department of Biomedical Engineering, The Fourth Military Medical University, Xi’an 710032, China; zhibozhao@fmmu.edu.cn (Z.Z.); daimeng@fmmu.edu.cn (M.D.); 2 Department of Aerospace Medicine, The Fourth Military Medical University, Xi’an 710032, China; 3 School of Biomedical Engineering, Guangzhou Medical University, Guangzhou 511436, China; zhanqizhao@gzhmu.edu.cn

**Keywords:** electrical impedance tomography, stimulation pattern, anti-noise performance, image interpretability

## Abstract

Pulmonary electrical impedance tomography (EIT) offers non-invasive and real-time imaging in a compact device size, making it valuable for pulmonary ventilation monitoring. However, conventional EIT stimulation patterns face a trade-off dilemma between anti-noise performance and image interpretability. To address this challenge, we propose a novel mixed stimulation pattern that integrates opposite and adjacent stimulation patterns with a tunable weight ratio. The results of simulations and human experiments (involving 30 subjects) demonstrated that the mixed stimulation pattern uses 200 stimulation–measurement channels, preserves a high signal-to-noise ratio, improves lung separation, and reduces artifacts compared with the opposite and adjacent stimulation patterns. It maintained stable imaging at 600 μA of stimulation current amplitude (equivalent to 1 mA) and preserved most imaging and clinical indicators’ stability at 200 μA (except GI/RVD_SD_). The adjustable weight ratio enables imaging performance to be flexibly adjusted according to different noise levels in acquisition environments. In conclusion, the pattern we proposed offers a superior alternative to traditional patterns, achieving a favorable balance of real-time capability, anti-noise performance, and image interpretability for pulmonary EIT imaging.

## 1. Introduction

Electrical Impedance Tomography (EIT) is sensitive to changes in electrical impedance within the imaging region. It reconstructs images of internal impedance changes by injecting electric current through surface electrodes and measuring the resulting voltages [1]. Changes in lung ventilation are accompanied by impedance variations inside the thoracic cavity, making EIT highly suitable for reconstructing internal ventilation images. EIT-based lung ventilation imaging offers advantages such as non-invasiveness, absence of ionizing radiation, capability for long-term monitoring, and low cost [2,3]. Leveraging these merits, EIT has been widely applied in the treatment and auxiliary diagnosis of respiratory diseases. Specific applications include evaluating lung recruitment [4,5], optimizing positive end-expiratory pressure (PEEP) titration [6], assessing ventilation-perfusion matching [7], guiding ventilator weaning [8,9], and evaluating pediatric respiratory rehabilitation [10]. Particularly during the COVID-19 pandemic, the clinical value of pulmonary EIT was further highlighted, achieving encouraging outcomes in the respiratory system repair and treatment of patients [11,12,13,14]. With its promising application prospects, pulmonary EIT has now entered the stage of clinical application [15].

As an imaging technology applied in clinical practice, the imaging stability and accuracy of EIT have attracted much attention. The signal quality of EIT is the foundation of stable imaging, and various image indicators of EIT are the characterization of imaging accuracy. The stimulation and measurement pattern is an important component of EIT and precisely one of the decisive factors for the signal quality and image quality of EIT. For pulmonary EIT systems, the opposite stimulation pattern and the adjacent stimulation pattern based on the four-electrode method are the most commonly used. To ensure the quality of EIT measurement signals, Adler et al. proposed an indicator characterizing the sensitivity of stimulation and measurement patterns to internal change measurements [16]. By comparing a series of traditional stimulation patterns, they found that the adjacent stimulation pattern, first proposed by Barber et al. [17] and now widely used, has the lowest measurement sensitivity—meaning the EIT signals collected under this pattern are most susceptible to interference—while the opposite stimulation pattern exhibits excellent detection capability. Additionally, Cheney et al. [18] and Bera et al. [19] independently validated the superior anti-noise performance of the opposite stimulation pattern through simulations and animal tissue phantom experiments, respectively. Building upon this, Tarabi et al. [20] conducted a detailed analysis of noise effects on EIT imaging quality, demonstrating that the opposite stimulation pattern yields more stable imaging capabilities. However, due to the low spatial resolution of EIT, in the opposite stimulation pattern, the left and right lung ventilation images in pulmonary EIT images often overlap, which has a large deviation from the actual ventilation status and seriously affects the imaging accuracy. To address this issue, some researchers have proposed the idea of lung-region constrained imaging [21], which leverages the characteristic that changes in thoracic impedance primarily occur within the lungs. Restricting image reconstruction to the lung region reduces reconstruction dimensions, thereby enhancing reconstruction accuracy. Both Bayesian-based [22] and discrete cosine transform-based [23] lung-region-constrained reconstruction algorithms achieve effective separation of left and right lung ventilation images. However, this method faces multiple limitations in practical applications. On one hand, this method needs to construct a lung-region constrained model for different patients and reset the image reconstruction matrix stored in the EIT device, which is not conducive to real-time and rapid imaging. On the other hand, impedance changes caused by cardiac ejection and respiratory motion are also constrained within the lung region, leading to significant errors. Surprisingly, the adjacent stimulation pattern has the advantage of a high lung separation degree and strong image interpretability. Russo et al. [24] found that while the opposite stimulation pattern exhibits strong noise resistance and central sensitivity, the adjacent stimulation pattern delivers superior performance for targets near electrodes, making it suitable for pulmonary imaging. Furthermore, adjacent stimulation provides more independent measurements, incorporating greater information during image reconstruction and thus offering enhanced image interpretability [25,26]. In general, the opposite stimulation pattern and adjacent stimulation pattern have their own unique advantages and disadvantages in pulmonary EIT imaging: the opposite stimulation pattern has strong anti-noise performance, but the left and right lung ventilation images are difficult to separate, leading to difficulties in image interpretation; the adjacent stimulation pattern yields lung ventilation images with better interpretability but is greatly affected by noise and has weaker imaging stability than the opposite stimulation pattern. Therefore, in practical applications, the use of EIT often faces the dilemma of choosing between anti-noise performance and image interpretability.

To address this problem and achieve enhanced image interpretability while ensuring signal quality, we propose a novel mixed stimulation pattern—this new stimulation and measurement pattern is formed by selecting and integrating channels with a high signal-to-noise ratio from the opposite stimulation pattern and channels with high sensitivity to the lung region from the adjacent stimulation pattern. To achieve deeper fusion, we further construct a weighted EIT image reconstruction algorithm for the mixed stimulation pattern. In addition, to evaluate the anti-noise performance and image interpretability of the mixed stimulation pattern, we conducted simulations and human experiments, respectively, to compare the signal quality and image indicators of the mixed stimulation pattern with those of the two traditional stimulation patterns. Finally, to assess the imaging stability of the mixed stimulation pattern, we carried out human experiments to compare the variation degrees of image and clinical indicators of the mixed stimulation pattern under different stimulation current amplitudes (1 mA, 600 μA, 200 μA).

The main contributions of this work are summarized as follows:

- Proposing a weight-adjustable mixed stimulation pattern to resolve the long-standing trade-off dilemma between anti-noise performance and image interpretability in traditional pulmonary EIT stimulation patterns.
- Achieving a balanced optimization of real-time capability, signal anti-noise performance, image interpretability, and artifact reduction.
- Providing a flexible trade-off parameter to improve EIT’s adaptability to diverse acquisition environments.
- Validating stable imaging in low-current scenarios, expanding the practical applicability of pulmonary EIT imaging.

## 2. Materials and Methods

### 2.1. Mixed Stimulation Pattern

The Skip-*n* pattern is a commonly used stimulation and measurement pattern in EIT systems [26], that is, a pattern where stimulation is applied to electrodes separated by *n* electrodes and measurements are taken on adjacent electrodes. For the 16-electrode EIT system most widely used in lung ventilation imaging, *n* = 7 corresponds to the opposite stimulation pattern (Skip-7), and *n* = 0 corresponds to the adjacent stimulation pattern (Skip-0). The former has the advantages of high measurement sensitivity, low noise interference, and stable lung ventilation images, while the latter has the advantage of strong image interpretability due to the high distinguishability between the left and right lungs in lung ventilation images. We integrated these two patterns and proposed a novel mixed stimulation pattern that combines high anti-noise performance and high interpretability.

#### 2.1.1. Measurement Channels of the Mixed Stimulation Pattern

EIT adopts the four-electrode method to measure boundary voltages. Due to the superposition theorem and reciprocity theorem of circuits, there are often a large number of redundant stimulation and measurement channels (SMCs) in the Skip-*n* pattern, i.e., SMCs that can be obtained by superposition or reciprocity of other SMCs. These redundant SMCs contribute nothing to image solving, but in practical applications, their existence is defaulted, considering the symmetry of stimulation and measurement. The number of SMCs in the opposite stimulation pattern is 192, and that in the adjacent stimulation pattern is 208. To avoid the decrease in acquisition speed and the increase in image solving cost caused by the doubling of the number of channels of the mixed stimulation pattern, the mixed stimulation pattern selects the independent channels of the opposite stimulation pattern and the adjacent stimulation pattern to complete its construction.

**Figure 1:**
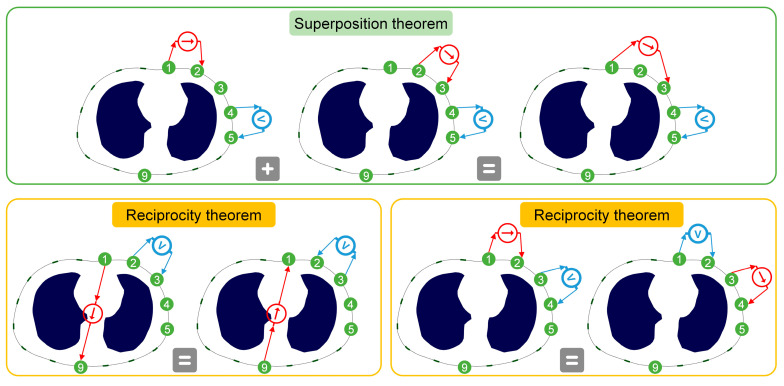
The generation process of redundant channels under the superposition and reciprocity theorems of circuits. The number indicates the electrode number, and the arrow indicates the direction of current injection or voltage measurement. The measurement channels following the “=” sign are redundant channels.

The superposition and reciprocity theorems of circuits influence the independence of measurement channels in stimulation patterns. Figure 1 visually illustrates the generation process of redundant channels under these theorems, encompassing three scenarios of redundancy. The opposite stimulation pattern involves 16 stimulations, with 12 measurements per stimulation, resulting in a total of 192 SMCs. According to the reciprocity theorem, swapping the polarity of the two electrodes in the stimulation electrode pair yields identical measurement results; thus, the latter half of the stimulations can be directly removed, retaining 96 SMCs. In fact, when both the superposition and reciprocity theorems are applied simultaneously, only 76 independent channels remain [27]. However, to ensure the symmetry of stimulation and measurement, the 20 redundant channels among the 96 channels are not removed. The adjacent stimulation pattern includes 16 stimulations, with 13 measurements per stimulation, giving a total of 208 SMCs. Owing to the reciprocity theorem, all SMCs corresponding to the first and second stimulations are fully independent, while each subsequent stimulation generates one additional redundant SMC. Therefore, there are 104 independent SMCs in this pattern. Ultimately, the mixed stimulation pattern comprises a total of 200 SMCs (the specific channel information is provided in Appendix A), maintaining the same acquisition speed and image solving cost as the opposite and adjacent stimulation patterns. Since the 96 opposite stimulation redundant channels and 104 adjacent stimulation redundant channels were identified solely based on the reciprocity theorem, as shown in Figure 1, each removed channel has a unique counterpart among the retained channels. This ensures that in subsequent EIT forward and inverse problem solutions, the removal of opposite and adjacent stimulation channels only alters the dimensionality of the sensitivity matrix without changing its rank and imaging characteristics. Consequently, the essential features of both stimulation patterns are fully preserved.

#### 2.1.2. EIT Image Reconstruction Algorithm for the Mixed Stimulation Pattern

The EIT linear reconstruction algorithm estimates the conductivity change $\Delta \hat{\sigma }$ by minimizing the mismatch between the actually measured boundary voltage change $y^{*}$ and the boundary voltage change, **y** (y=JΔσ, where **J** is the sensitivity matrix), induced by the conductivity change, Δσ,

(1) $$\begin{array}{cc} \Delta \hat{\sigma } & =\arg\underset{\Delta \sigma }{\min}[{\left\|W(J\Delta \sigma -y*)\right\|}^{2}+\lambda^{2}{\left\|\Gamma \Delta \sigma \right\|}^{2}]\\ & ={\left(J^{T}W^{T}WJ+\lambda^{2}\Gamma^{T}\Gamma \right)}^{-1}J^{T}W^{T}Wy*\\ & ={\left(\Gamma^{T}\Gamma \right)}^{-1}J^{T}{\left(J{\left(\Gamma^{T}\Gamma \right)}^{-1}J^{T}+\lambda^{2}{\left(W^{T}W\right)}^{-1}\right)}^{-1}y*, \end{array}$$

where **W** is a diagonal matrix whose diagonal elements are the weights of the corresponding SMCs; Γ is a regularization matrix; and $\lambda^{2}$ is a regularization parameter. Γ is set as an identity matrix to impose regularization constraints.

Building upon the above, we develop an EIT image reconstruction algorithm for the mixed stimulation pattern. The weights of SMCs from the opposite stimulation pattern and adjacent stimulation pattern are set as *w*_op_ and *w*_ad_, respectively. Meanwhile, **J** and **y** of the mixed stimulation pattern are decomposed into **J**_op_, **J**_ad_, $y_{\mathrm{op}}^{*}$, and $y_{\mathrm{ad}}^{*}$ corresponding to the two patterns. Then, Equation (1) can be rewritten as

(2) $$\Delta \hat{\sigma }={\left[\begin{array}{c} J_{\mathrm{op}}\\ J_{\mathrm{ad}} \end{array}\right]}^{T}{\left(\left[\begin{array}{c} J_{\mathrm{op}}\\ J_{\mathrm{ad}} \end{array}\right]{\left[\begin{array}{c} J_{\mathrm{op}}\\ J_{\mathrm{ad}} \end{array}\right]}^{T}+\lambda^{2}{\left[\begin{array}{cc} w_{\mathrm{op}}^{2}I_{\mathrm{op}} & 0\\ 0 & w_{\mathrm{ad}}^{2}I_{\mathrm{ad}} \end{array}\right]}^{-1}\right)}^{-1}\left[\begin{array}{c} y_{\mathrm{op}}^{*}\\ y_{\mathrm{ad}}^{*} \end{array}\right],$$

where **I**_op_ and **I**_ad_ are identity matrices. According to Equation (2), the conductivity estimation under the mixed stimulation pattern is actually a fusion of the estimation results of the opposite stimulation pattern and the adjacent stimulation pattern, and thus is expected to have both the advantages of a high signal-to-noise ratio (SNR) and strong interpretability. To establish a neutral, unbiased benchmark model that directly demonstrates the inherent advantages of the new method framework over traditional approaches, we set *w*_op_ equal to *w*_ad_ for subsequent experiments to eliminate potential bias stemming from initial weight distribution. However, it is worth mentioning that different proportional relationships between *w*_op_ and *w*_ad_ may yield better fusion effects.

The GREIT algorithm is currently the most widely used linear reconstruction algorithm for lung ventilation imaging [28]. We constructed the desired image matrix **D** based on EIT consensus indicators [29], converted the conductivity estimation into the desired EIT image, and then obtained the image reconstruction matrix **R** of GREIT defined from distributions [30]:

(3) $$R=DJ^{T}{\left(J^{T}J+\lambda^{2}I\right)}^{-1}.$$

Finally, the GREIT-reconstructed image **x** of the mixed stimulation pattern is derived:

(4) $$x=Ry^{*}.$$

### 2.2. Performance Evaluation of the Mixed Stimulation Pattern via Simulation and Human Experiments

**Figure 2:**
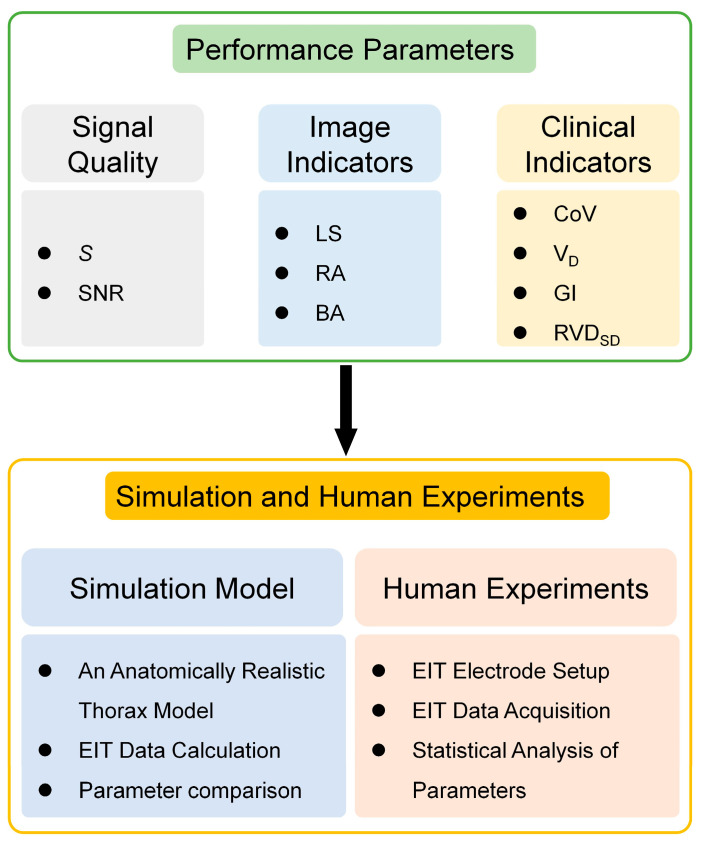
Performance evaluation flowchart of the mixed stimulation pattern. “*S*” is the measurement sensitivity. “SNR” is the EIT signal signal-to-noise ratio. “LS” is the lung separation. “RA” are the ringing reverse artifacts. “BA” are the boundary artifacts. “CoV” is the center of ventilation. “V_D_” is the dorsal fraction of ventilation. “GI” is the global inhomogeneity. “RVD_SD_” is the regional ventilation delay’s standard deviation. These performance parameters are detailed in Section 2.2.1.

The mixed stimulation pattern integrates the opposite stimulation and adjacent stimulation, and has the potential to combine the common advantages of the two patterns (strong anti-noise performance and high image interpretability). We designed precise simulation experiments and human experiments to evaluate the performance of the mixed stimulation pattern by comparing it with the two traditional stimulation patterns. In addition, in human experiments, we analyzed the stability of the mixed stimulation pattern by comparing the changes in performance parameters of the mixed stimulation pattern under different stimulation current amplitudes. The flowchart of the evaluation process is shown in Figure 2.

#### 2.2.1. Performance Parameters

1. Measurement Sensitivity and EIT Signal SNR

Measurement sensitivity reflects the detection capability of different stimulation patterns for the same impedance change; the stronger the detection capability, the better the anti-noise performance. For simulation experiments, due to the lack of process information on lung ventilation, only the differences between end-inspiration and end-expiration are generally considered. Therefore, we compare the anti-noise performance of stimulation patterns through measurement sensitivity. Among them, the definition of the measurement sensitivity *S* of a stimulation pattern is as follows:

(5) $$S=\frac{1}{N}\sum_{c}^{N}\left|\Delta V_{c}\right|,$$

where *N* is the number of measurement channels included in the stimulation pattern, and Δ*V_c_* is the difference in measured voltage of the *c*-th channel between end-inspiration and end-expiration.

**Figure 3:**
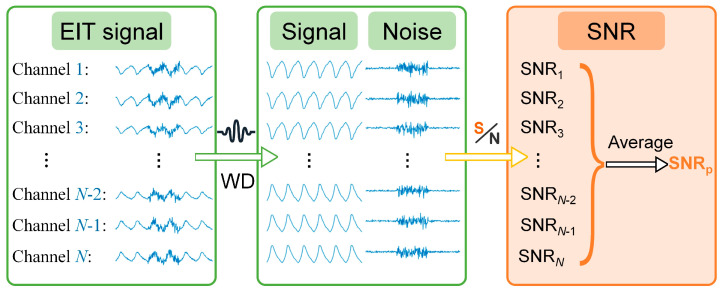
Calculation method of SNR for multi-channel EIT signals. “*N*” represents the number of measurement channels of the stimulation pattern, “WD” represents wavelet decomposition, and “S/N” represents the calculation of SNR.

SNR takes into account noise interference throughout the entire lung ventilation process, reflecting the relationship between the measured signal and environmental noise, and can fully evaluate the anti-noise performance of stimulation patterns. Therefore, for human experiments, we compare the anti-noise performance of stimulation patterns through SNR. The calculation method of the SNR of pulmonary EIT signals is as follows [31]: obtain the time-series signal of the measured voltage of each SMC (with a total length of *T*), apply wavelet decomposition (the wavelet basis function is symlets, the order is 7, and the decomposition level is 3) to separate the signal *s*(*t*) from the noise *n*(*t*) (where *t* = 0, 1, …, *T* − 1), and calculate the channel SNR (SNR*_c_*). The average value of the SNR of all channels included in the stimulation pattern is the corresponding SNR (SNR_p_) of the pattern (Figure 3 shows the calculation flow chart of SNR_p_):

(6) $$\left\{\begin{array}{l} {\mathrm{SNR}}_{c}=10\log_{10}\frac{\sum_{t=0}^{T-1}{\left|s(t)\right|}^{2}}{\sum_{t=0}^{T-1}{\left|n(t)\right|}^{2}}\\ {\mathrm{SNR}}_{p}=\frac{1}{N}\sum_{c}^{N}{\mathrm{SNR}}_{c} \end{array}\right..$$

2. Lung Ventilation EIT Image Indicators

Image interpretability is one of the most concerning topics in medical imaging technologies. For pulmonary EIT imaging, the degree of left-right lung separation, ringing reverse artifacts, and boundary artifacts are key indicators affecting the interpretability of EIT images [32].

**Figure 4:**
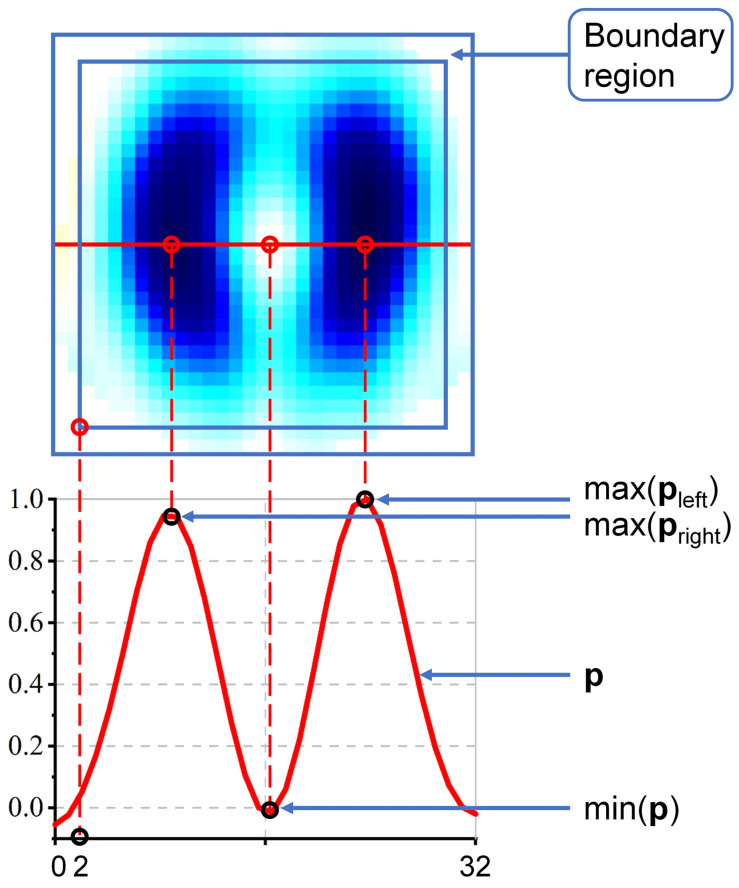
Key parameters for the calculation of lung separation (LS) and boundary artifacts (BA).

Due to the low spatial resolution of EIT, EIT images may present features of overlapping ventilation between the left and right lungs, which has an obvious and intuitive conflict with the real lung ventilation distribution and seriously affects the interpretability of EIT images. Therefore, accurate lung separation (LS) is desirable (generally, the larger the value, the better). The calculation method of LS is as follows:

(7) $$\mathrm{LS}=\frac{\max(p_{\mathrm{left}})-\min(p)}{\max(p_{\mathrm{left}})}+\frac{\max(p_{\mathrm{right}})-\min(p)}{\max(p_{\mathrm{right}})},$$

where **p** denotes the sequence of pixel values corresponding to the coronal centerline in the pulmonary ventilation EIT image, while **p**_left_ and **p**_right_ represent the vectors composed of pixels located to the left and right of the sagittal line in **p**, respectively. Figure 4 illustrates the computational parameters for LS.

Ringing reverse artifacts (RA) originate from L2 regularization during the reconstruction process, as L2 regularization excessively suppresses high-frequency components, which in turn leads to the Gibbs phenomenon (i.e., RA in EIT images) [33]. In pulmonary EIT images, positive/negative pixel values indicate increased/decreased impedance. Therefore, RA caused by non-physiological changes can lead to misjudgment of the actual impedance changes in the chest region, resulting in difficulties in image interpretation. A small RA value is desirable. The calculation method of RA is as follows:

(8) $$\mathrm{RA}=\sum_{{\left[x\right]}_{i}<0}\left|{\left[x\right]}_{i}\right|/\sum_{{\left[x\right]}_{i}>0}{\left[x\right]}_{i},$$

where ${\left[x\right]}_{i}$ is the value of the *i*-th pixel of the reconstructed image **x**.

Boundary artifacts (BA) refer to the erroneous estimation of impedance changes generated at the boundary region of reconstructed images. During the lung ventilation process, large impedance changes are caused by pulmonary respiration, and small impedance changes are induced by cardiac ejection, both of which occur inside the imaging region. Therefore, the estimated impedance changes in the boundary region obtained during the reconstruction of lung ventilation images are inconsistent with the actual situation and are classified as imaging errors. These errors may arise from respiratory body movement, changes in contact impedance between electrodes and skin, or other unknown factors (such influencing factors do not exist in simulation data, so this parameter is not discussed in simulation experiments), and affect image interpretation. A small BA value is desirable. The calculation method of BA is as follows:

(9) $$\mathrm{BA}=\sum_{{\left[x\right]}_{i}\in B}\left|{\left[x\right]}_{i}\right|/\sum_{{\left[x\right]}_{i}>0}{\left[x\right]}_{i},$$

where *B* is the boundary region of the reconstructed image (the boundary region is marked in Figure 4). The three parameters, LS, RA, and BA, are all calculated based on the EIT tidal breathing image (the lung impedance change image at the end-inspiration moment with the end-expiration moment as the reference).

3. Lung Ventilation EIT Clinical Indicators

For lung ventilation imaging, in addition to image indicators, the accuracy of clinical indicators is also our key focus. The larger the stimulation current, the less the interference from environmental noise, and the more accurate the indicators. Yang et al. pointed out that the opposite stimulation pattern maintains a certain level of accuracy at 250 μA, while the adjacent stimulation pattern requires a current of more than 500 μA [30]. Based on the image indicators and clinical indicators calculated from human EIT data under three stimulation current amplitudes (1 mA, 600 μA, and 200 μA), we evaluated the stability of the image indicators and clinical indicators of the mixed stimulation pattern under low-current conditions. Four key EIT-based clinical indicators were selected, including center of ventilation (CoV), dorsal fraction of ventilation (V_D_), global inhomogeneity (GI), and regional ventilation delay’s standard deviation (RVD_SD_) [34].

#### 2.2.2. Details of the Simulation Model

**Figure 5:**
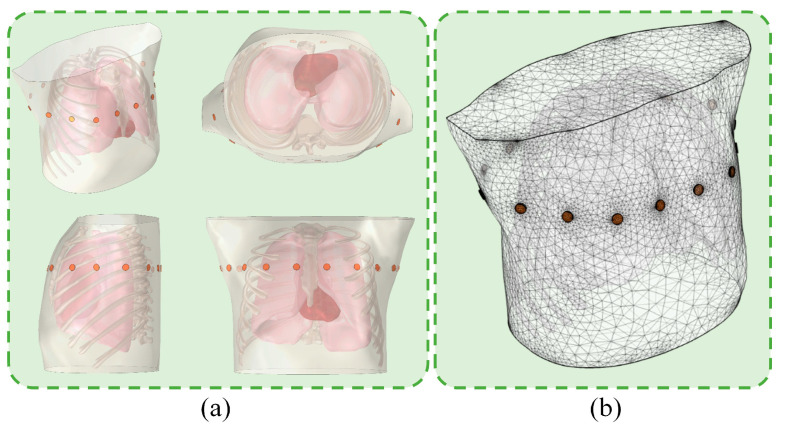
Accurate thoracic finite element model. (**a**) Geometric information of the thoracic model, including the axonometric view, bottom view, side view, and front view. (**b**) Finite element mesh of the thoracic model, which includes a total of 34,228 nodes and 183,614 elements.

We constructed a complete 3D thoracic finite element model (Figure 5) by distinguishing different tissue regions, setting EIT electrodes, and configuring the electrical conductivity of thoracic tissues [31]. This 3D thoracic model includes six tissue regions that significantly affect EIT measurements, namely the lungs, heart, spine, ribs, sternum, and trachea, with the boundaries of these tissues derived from real CT images. In addition, a skin layer with a thickness of approximately 2 mm and a fat layer of 1 mm were set in the peripheral area of the model, and the remaining area was filled with muscle tissue. A double-layer electrode model consisting of metal electrode patches and conductive gel was used to achieve an accurate simulation of EIT electrodes. Sixteen electrodes with a radius of 5 mm were placed at equal intervals on the horizontal plane between the fourth and fifth intercostal spaces.

Simulated signals were obtained by solving the EIT forward problem based on this model: the boundary conditions of the finite element model were set (applying source or sink current to the stimulation electrodes), with an electric field existing in the model region, and the potential distribution u(r) in the region satisfied the Laplace equation and was constrained by the set boundary conditions:

(10) $$\nabla \left(\sigma (r)\nabla u(r)\right)=0,\;\;\;\;r\in \Omega ,$$

(11) $$\int_{e_{i}}\sigma (r)\frac{\partial u(r)}{\partial n}dS=I_{i},\;\;\;\;r\in e_{i},i\in 1,\ldots ,16,$$

(12) $$\sigma (r)\frac{\partial u(r)}{\partial n}=0,\;\;\;\;r\in \partial \Omega \backslash \overset{16}{\underset{i=1}{\cup }}e_{i},$$

where *e_i_* is the domain of the *i*-th electrode, *I_i_* is the current of the *i*-th electrode, σ(r) is the electrical conductivity distribution of the model, and **n** is the outward unit normal vector of the stimulation electrode. The full electrode model [35] was applied:

(13) $$u(r)+Z_{i}\sigma (r)\frac{\partial u(r)}{\partial n}=U_{i},\;\;\;\;r\in e_{i},i\in 1,\ldots ,16,$$

where *Z_i_* is the contact impedance between the stimulation electrode and the skin, and *U_i_* is the potential of the *i*-th electrode. Set a ground point and combine Equations (10)–(13), and then the Galerkin finite element method can be applied to solve for u(r) and the potential of the measurement electrodes. Then, the voltage was calculated through adjacent measurements, and the voltages of all SMCs of different stimulation patterns could be obtained by switching the stimulation electrodes.

#### 2.2.3. Human Experiment Protocol

Ethical Statement: This study protocol has been approved by the Ethics Committee of the Fourth Military Medical University, Xi’an, Shaanxi Province, People’s Republic of China (KY20253789-1). The study was conducted in accordance with the principles embodied in the Declaration of Helsinki and local legal requirements. All subjects were informed of the study protocol and signed written informed consent prior to participating in the study.

Fifteen healthy adult male subjects and fifteen healthy adult female subjects participated in this experiment, and the experimental details are as follows:

- EIT Electrode Setup: In accordance with expert consensus, the 16-electrode belt was placed on the horizontal plane of the 4th–5th intercostal space of the subjects’ chest. The 16 electrodes were distributed equidistantly in a clockwise direction (from the foot-to-head view), with electrode 1 and electrode 16 located on the left and right sides of the median sagittal plane of the human body, respectively.
- EIT Data Acquisition Equipment: We used a wireless wearable electrical impedance tomography system to collect EIT data from 30 subjects. The current stimulation frequency of this system is 50 kHz, the sampling rate is 20 frames/s, and the current range is 100 μA to 1 mA. The results of resistance phantom tests showed that the measurement SNR of the device is greater than 70 dB, and the relative change in measurement within 3 h is less than 0.1% [36].
- EIT Data Acquisition Process: During the entire data acquisition period, subjects were required to maintain a sitting posture and breathe calmly. Opposite stimulation and adjacent stimulation were selected, respectively, with a stimulation current amplitude of 1 mA set. In addition, the mixed stimulation pattern was selected, and three stimulation current amplitudes (1 mA, 600 μA, and 200 μA) were configured in sequence. There were a total of 5 acquisition configurations above, and for each configuration, EIT data were collected for more than 2 min.
- Statistical Analysis: Data from male and female subjects were analyzed independently, with 15 sets of data for each gender. For the pulmonary EIT signals collected for more than 2 min under the three stimulation patterns with a stimulation current amplitude of 1 mA, their signal SNR and image indicators were calculated. In addition, for the pulmonary EIT signals collected for more than 2 min under the mixed stimulation pattern with three stimulation current amplitudes (1 mA, 600 μA, and 200 μA), their image indicators and clinical indicators were calculated. First, normality test and homogeneity of variance test were performed. On this basis, traditional ANOVA was applied to compare the differences in signal SNR and image indicators among different stimulation patterns (aiming to evaluate the anti-noise performance and imaging capability of the mixed stimulation pattern), and to compare the differences in image indicators and clinical indicators among different current amplitudes (aiming to evaluate the stability of the mixed stimulation pattern under low-current conditions), with Tukey’s HSD test used for multiple comparisons. In addition, for data that failed the homogeneity of variance test, Welch’s ANOVA and Games–Howell test were used for multiple comparisons. For data that failed the normality test, the Friedman test was used for analysis, and pairwise comparisons were conducted via the corrected paired Wilcoxon signed-rank test.

## 3. Results

To evaluate the performance of the mixed stimulation pattern, we conducted two comparative experiments. On one hand, to verify its anti-noise performance and imaging performance, we performed precise simulations and human experiments to compare the mixed stimulation pattern with two traditional stimulation patterns. The parameters reflecting anti-noise performance, measurement sensitivity and SNR_p_ were applied to simulation experiments (noise-free scenarios) and human experiments (noise-containing scenarios), respectively. The parameters reflecting imaging performance were LS, RA, and BA. On the other hand, to evaluate the stability of the mixed stimulation pattern’s imaging indicators and clinical indicators for pulmonary ventilation, we conducted human experiments comparing the mixed stimulation pattern at different current amplitudes. The imaging indicators were LS, RA, and BA, while the clinical indicators were CoV, VD, GI, and RVD_SD_. The simulation and human experiments are described separately below.

### 3.1. Simulation Experiments

**Figure 6:**
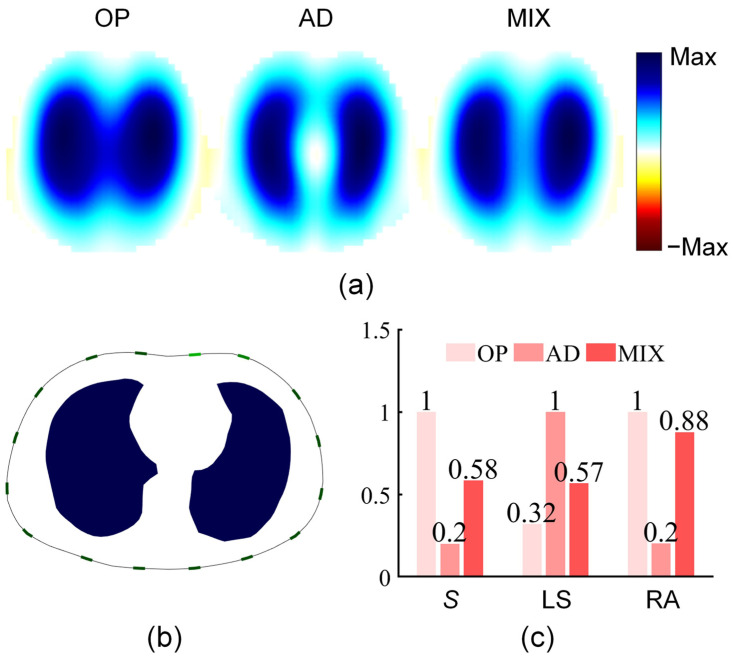
Results of simulation experiments. (**a**) Pulmonary ventilation images of opposite stimulation pattern (OP), adjacent stimulation pattern (AD), and mixed stimulation pattern (MIX). (**b**) Actual distribution of lung ventilation in the electrode plane. (**c**) Comparison of measurement sensitivity (*S*), lung separation (LS), and ringing reverse artifacts (RA).

To theoretically evaluate the anti-noise performance and image interpretability of the mixed stimulation pattern, we set the lung conductivity at end-inspiration and end-expiration based on an accurate thoracic finite element model, and calculated the noise-free boundary voltages and EIT lung ventilation images under the three stimulation patterns. Measurement sensitivity *S* and two image indicators (LS and RA) were calculated based on the boundary voltages and lung ventilation images, respectively, with the results shown in Figure 6.

The experimental results indicated that compared with the two traditional stimulation patterns, the mixed stimulation pattern exhibited compromised performance across the three performance parameters. Specifically, for *S*, the mixed stimulation was weaker than the opposite stimulation, but showed a significant improvement compared with adjacent stimulation, with its measurement sensitivity being approximately three times that of adjacent stimulation. A high *S* value reflects the strong anti-noise performance of the mixed stimulation pattern. For LS and RA, the mixed stimulation was inferior to adjacent stimulation, but was significantly better than opposite stimulation, especially achieving about a twofold improvement in LS (the left and right lung ventilation images of mixed stimulation in Figure 6a have been separated).

### 3.2. Human Experiments

#### 3.2.1. Comparison of Three Stimulation Patterns

**Figure 7:**
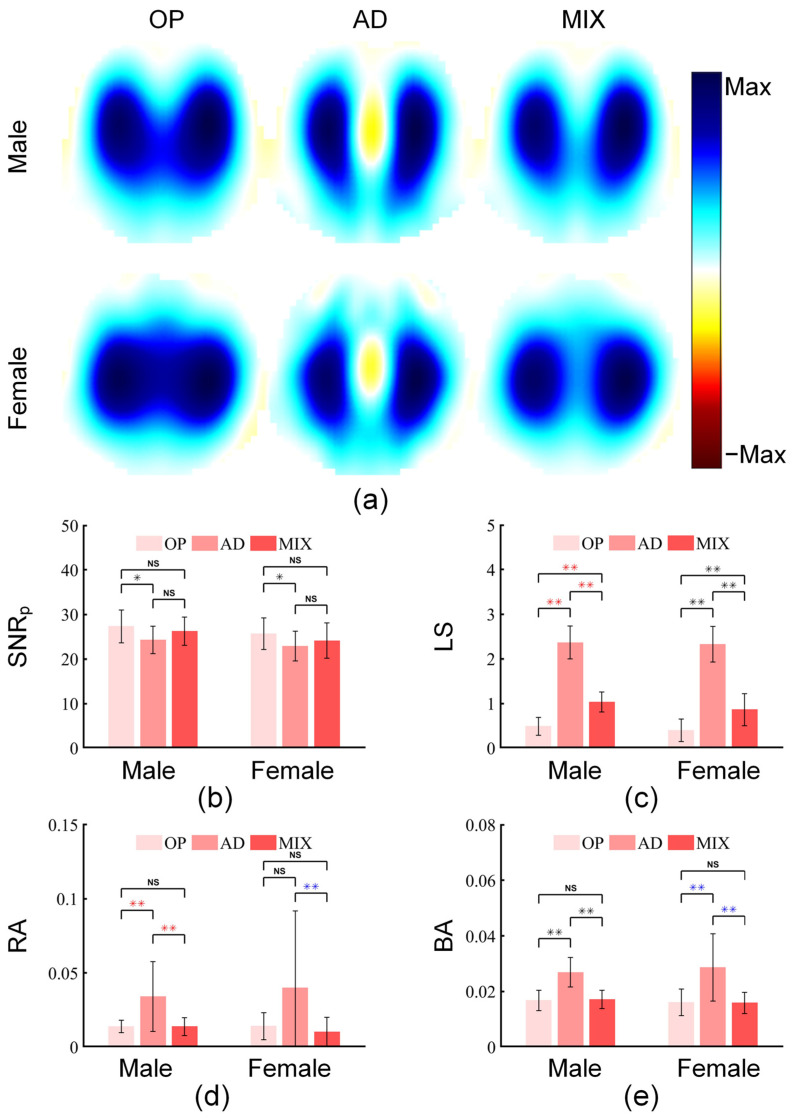
Comparison of SNR and image indicators among different stimulation patterns. (**a**) Pulmonary ventilation images of opposite stimulation pattern (OP), adjacent stimulation pattern (AD), and mixed stimulation pattern (MIX), derived from one male subject and one female subject. (**b**–**e**) show the comparisons of the three stimulation patterns in terms of SNR_p_, lung separation (LS), ringing reverse artifacts (RA), and boundary artifacts (BA), respectively. “NS” indicates no significant difference between two groups of data; “*” indicates a significant difference between two groups of data with *p* < 0.05; “**” indicates a significant difference between two groups of data with *p* < 0.01. Black asterisks represent the results of Tukey’s HSD test for data that meet the requirements of normality and homogeneity of variance; red asterisks represent the results of the Games–Howell test for data that do not meet the requirement of homogeneity of variance; blue asterisks represent the results of corrected paired Wilcoxon signed-rank test for data that meet neither the normality nor the homogeneity of variance requirements.

To evaluate the anti-noise performance and image interpretability of the mixed stimulation pattern using real human data (containing noise), based on the experimental dataset of 30 subjects (each dataset includes EIT signals collected for more than two minutes under three stimulation patterns), we calculated the SNR and image indicators of these signals. Figure 7 shows the comparison of SNR_p_ and three image indicators (LS, RA, and BA) among the mixed stimulation pattern and the two traditional stimulation patterns.

The results showed that for the SNR_p_ of both male and female groups, the opposite stimulation was significantly higher than the adjacent stimulation (*p* < 0.05), while there were no significant differences between mixed stimulation and opposite stimulation, nor between mixed stimulation and adjacent stimulation. However, the mean value of mixed stimulation was close to that of opposite stimulation and higher than that of adjacent stimulation, indicating that the SNR_p_ of mixed stimulation maintained a high level.

For the LS of both male and female groups, adjacent stimulation was significantly higher than opposite stimulation and mixed stimulation (*p* < 0.01, *p* < 0.01), and mixed stimulation was also significantly higher than opposite stimulation (*p* < 0.01), which demonstrated the improvement of mixed stimulation in LS.

For the RA of the male group, adjacent stimulation was significantly higher than opposite stimulation and mixed stimulation (*p* < 0.01, *p* < 0.01), and no significant difference was found between opposite stimulation and mixed stimulation. For the RA of the female group, due to the obvious variability of adjacent stimulation, although its mean value was much higher than that of opposite stimulation, no significant difference was observed; in contrast, mixed stimulation was significantly lower than adjacent stimulation (*p* < 0.01). These results reflected the advantage of mixed stimulation in RA.

For the BA of both male and female groups, adjacent stimulation was significantly higher than opposite stimulation and mixed stimulation (*p* < 0.01, *p* < 0.01), and opposite stimulation was similar to mixed stimulation, which manifested the advantage of mixed stimulation in BA.

#### 3.2.2. Comparison of the Mixed Stimulation Pattern Under Different Stimulation Current Amplitudes

**Figure 8:**
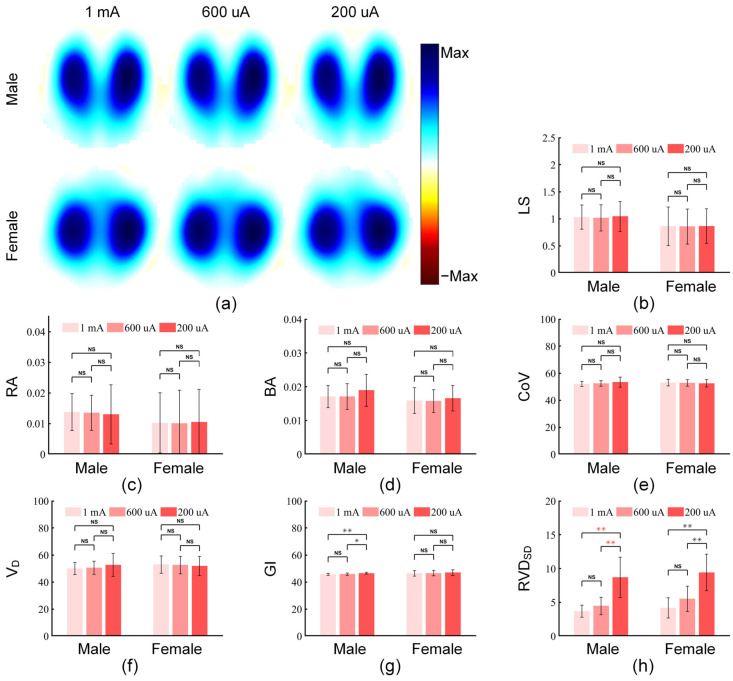
Comparison of image indicators and clinical indicators of the mixed stimulation pattern under different stimulation current amplitudes. (**a**) Pulmonary ventilation images of the mixed stimulation pattern at stimulation current amplitudes of 1 mA, 600 μA, and 200 μA. (**b**–**h**) show the comparisons of the three current amplitudes in terms of lung separation (LS), ringing reverse artifacts (RA), boundary artifacts (BA), center of ventilation (CoV), dorsal fraction of ventilation (VD), global inhomogeneity (GI), and regional ventilation delay’s standard deviation (RVD_SD_), respectively. “NS” indicates no significant difference between two groups of data; “*” indicates a significant difference between two groups of data with *p* < 0.05; “**” indicates a significant difference between two groups of data with *p* < 0.01. Black asterisks represent the results of Tukey’s HSD test for data that meet the requirements of normality and homogeneity of variance; red asterisks represent the results of the Games–Howell test for data that do not meet the requirement of homogeneity of variance; blue asterisks represent the results of corrected paired Wilcoxon signed-rank test for data that meet neither the normality nor the homogeneity of variance requirements.

To evaluate the stability of image indicators and clinical indicators of the mixed stimulation pattern under different stimulation current amplitudes and analyze its performance in low-current scenarios, based on the experimental dataset of 30 subjects (each dataset includes EIT signals collected for more than two minutes under the mixed stimulation pattern with three stimulation current amplitudes: 1 mA, 600 μA, and 200 μA), we calculated the image indicators and clinical indicators for these signals. Figure 8 shows the comparison of three image indicators and four pulmonary ventilation EIT clinical indicators (CoV, VD, GI, and RVD_SD_) of the mixed stimulation pattern under different stimulation current amplitudes.

The results showed that for the three image indicators (LS, RA, and BA) and two clinical indicators (CoV and VD) in both male and female groups, no significant differences were found among the three stimulation current amplitudes, indicating that the mixed stimulation pattern can maintain stability in most key indicators even at a low current of 200 μA.

For the GI of the male group, the current amplitude of 200 μA was significantly different from those of 1 mA and 600 μA (*p* < 0.01, *p* < 0.05), while in the female group, the GI values under the three current amplitudes were similar. For the RVD_SD_ of both male and female groups, the current amplitude of 200 μA showed significant differences from those of 1 mA and 600 μA (*p* < 0.01, *p* < 0.01).

Overall, compared with the current amplitude of 1 mA, all image and clinical indicators of the current amplitude of 600 μA maintained consistent stability; all image indicators under the current amplitude of 200 μA maintained consistent stability, but some clinical indicators (GI and RVD_SD_) showed variability.

## 4. Discussion

In this study, based on the classic opposite and adjacent stimulation patterns, we proposed a new EIT stimulation pattern for pulmonary ventilation imaging—the mixed stimulation pattern, which aims to integrate the respective advantages of high anti-noise performance and high interpretability of the two classic stimulation patterns. Through accurate simulation experiments and well-designed human experiments, we evaluated the anti-noise performance and image interpretability of the mixed stimulation pattern on the one hand, and tested the stability of the mixed stimulation pattern in low-current scenarios on the other hand.

### 4.1. Analysis of Simulation and Human Experiment Results

In the results of simulation experiments (i.e., under the noise-free condition), the mixed stimulation pattern exhibited intermediate performance in both measurement sensitivity and image indicators between the opposite stimulation and adjacent stimulation, and, in particular, achieved a balance between *S* and LS. In the results of human experiments comparing different stimulation patterns (i.e., under the noisy condition), although the LS of the mixed stimulation pattern was inferior to that of adjacent stimulation, it was significantly better than that of opposite stimulation, and its other parameters showed excellent performance among the three stimulation patterns. Therefore, we recommend selecting the mixed stimulation pattern to replace the traditional opposite stimulation and adjacent stimulation. In addition, the RA of adjacent stimulation was the largest in human experiments, which was contrary to the simulation results. This indicates that in real-scenario imaging, noise severely affects the imaging, and thus anti-noise performance can be regarded as a key indicator for evaluating stimulation patterns.

In the human experiments comparing the mixed stimulation pattern under different stimulation current amplitudes, most performance parameters remained stable across the three current amplitudes; however, the low current of 200 μA showed significant differences from high currents in GI and RVD_SD_, especially in RVD_SD_. Unlike CoV, VD, and GI (which only focus on impedance changes between end-inspiration and end-expiration), RVD_SD_ is a procedural indicator. Therefore, it is necessary to consider EIT images at moments when the lungs inhale a small amount of gas and the impedance change is small. At such times, low currents are highly susceptible to environmental noise, which can easily lead to errors. Hence, we suggest that the stimulation current amplitude should be greater than 200 μA when using the mixed stimulation pattern for pulmonary ventilation imaging.

In the human experiments, we analyzed male and female subjects separately. In the comparisons of different stimulation patterns and current amplitudes, the male and female groups showed consistency in most aspects, while a few differences are worth discussing.

Regarding the three stimulation patterns: In the male group, RA of opposite stimulation (OP) and adjacent stimulation (AD) exhibited a significant difference (*p* < 0.01) with a large effect size (Cohen’s d = −1.2259). However, in the female group, no significant difference was observed in RA between OP and AD (*p* > 0.05) with a moderate effect size (r = 0.41). The significant *p*-value and large effect size in males support the conclusion of practically meaningful differences between OP and AD. In females, the non-significant *p*-value indicates insufficient evidence to reject the null hypothesis of no difference, but the moderate effect size (r = 0.41) suggests caution—this may stem from inadequate statistical power (high within-group variability) rather than true equivalence.

Regarding different stimulation current amplitudes: In the male group, GI showed significant differences with large effect sizes between 1 mA and 200 μA (*p* < 0.01, Cohen’s d = −1.112) and between 600 μA and 200 μA (*p* < 0.05, Cohen’s d = −0.945). In contrast, the female group exhibited no significant differences in GI for these comparisons, with small effect sizes (1 mA vs. 200 μA: *p* > 0.05, Cohen’s d = −0.242; 600 μA vs. 200 μA: *p* > 0.05, Cohen’s d = −0.226). The significant *p*-values and large effect sizes in males strongly indicate that a low stimulation current (200 μA) indeed causes substantial changes in GI, whereas GI remained relatively stable at low currents in females.

These gender-specific differences suggest that sex is likely an important factor influencing EIT parameters, highlighting the need for exploring gender-specific evaluation criteria in future research.

### 4.2. Weighted Mixed Stimulation Pattern

In this study, the proposed mixed stimulation pattern adopted a 1:1 weight ratio to integrate the opposite stimulation and adjacent stimulation, achieving favorable imaging results; however, the 1:1 weight ratio is not necessarily the optimal choice. As can be seen from Equations (1) and (3), the weighted GREIT reconstruction matrix is

(14) $$R=DJ^{T}{\left(JJ^{T}+\lambda^{2}{\left(W^{T}W\right)}^{-1}\right)}^{-1}.$$

We performed the following transformation to obtain

(15) $$\begin{array}{cc} R & =DJ^{T}{\left(JJ^{T}+\lambda^{2}{\left(W^{T}W\right)}^{-1}\right)}^{-1}\\ & =DJ^{T}W^{T}{\left(W^{T}\right)}^{-1}{\left(JJ^{T}+\lambda^{2}{\left(W^{T}W\right)}^{-1}\right)}^{-1}W^{-1}W\\ & =DJ^{T}W^{T}{\left(WJJ^{T}W^{T}+\lambda^{2}I\right)}^{-1}W\\ & =D{\left(WJ\right)}^{T}{\left(WJ{\left(WJ\right)}^{T}+\lambda^{2}I\right)}^{-1}W, \end{array}$$

At this time, the GREIT reconstruction matrix of the weighted mixed stimulation pattern is

(16) $$R=D{\left[\begin{array}{c} w_{\mathrm{op}}^{2}J_{\mathrm{op}}\\ w_{\mathrm{ad}}^{2}J_{\mathrm{ad}} \end{array}\right]}^{T}{\left(\left[\begin{array}{c} w_{\mathrm{op}}^{2}J_{\mathrm{op}}\\ w_{\mathrm{ad}}^{2}J_{\mathrm{ad}} \end{array}\right]{\left[\begin{array}{c} w_{\mathrm{op}}^{2}J_{\mathrm{op}}\\ w_{\mathrm{ad}}^{2}J_{\mathrm{ad}} \end{array}\right]}^{T}+\lambda^{2}I\right)}^{-1}\left[\begin{array}{cc} w_{\mathrm{op}}^{2}I_{\mathrm{op}} & 0\\ 0 & w_{\mathrm{ad}}^{2}I_{\mathrm{ad}} \end{array}\right],$$

where Equation (16) is equivalent to Equation (2). It can be inferred from Equations (1), (4) and (16) that the reconstruction of the weighted mixed stimulation pattern is essentially a weighting of the sensitivity matrix **J** and the boundary voltage y*. Therefore, the GREIT defined from training sets can be easily implemented based on Equation (16) [30].

**Figure 9:**
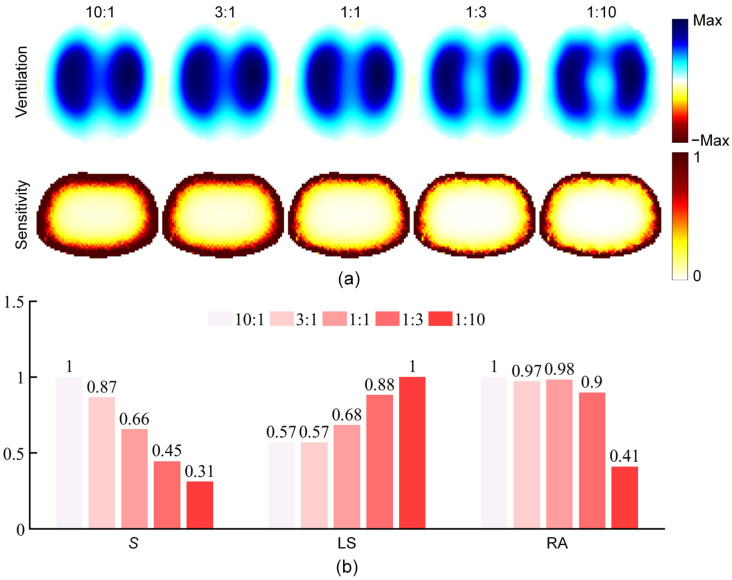
Imaging characteristics of the mixed stimulation pattern based on different weight ratios. (**a**) Pulmonary ventilation images and sensitivity distribution images (normalized by maximum value) when the weight ratio of opposite stimulation to adjacent stimulation is 10:1, 3:1, 1:1, 1:3, and 1:10, respectively. (**b**) Comparison of the mixed stimulation pattern with five weight ratios in terms of measurement sensitivity (*S*), lung separation (LS), and ringing reverse artifacts (RA).

To further test the characteristics of pulmonary ventilation images of the weighted mixed stimulation pattern, we set five different weight ratios, *w*_op_:*w*_ad_, as 10:1, 3:1, 1:1, 1:3, and 1:10, respectively. Based on the thoracic simulation model, the imaging results, sensitivity distribution in the imaging domain, measurement sensitivity, and image indicators under different weight ratios were obtained (Figure 9). The sensitivity distribution showed that the sensitivity of the central region decreased with the reduction in the weight ratio. During the EIT image reconstruction process, due to the addition of the regularization term, the imaging amplitude response in the imaging domain is affected by the sensitivity distribution [37]. At a consistent regularization level, different sensitivity distributions lead to inconsistent LS of pulmonary ventilation images. Therefore, fundamentally, the mixed stimulation pattern achieves image enhancement by adjusting the sensitivity distribution. The imaging results indicated that under the weight ratio of 10:1, the left and right lung ventilation images overlapped; under the weight ratio of 1:10, the left and right lung ventilation images showed a certain degree of excessive separation. Therefore, it is necessary to further discuss the selection of the weight ratio to achieve the optimal LS.

In addition, both *S* and LS of the mixed stimulation pattern exhibit obvious and stable correlations with the weight ratio. Therefore, in practical imaging, on the one hand, the weight ratio can be used as a parameter to dynamically balance the two performance indicators according to the on-site data acquisition conditions. For example, in scenarios where the signal acquisition quality is significantly excellent, the weight ratio can be reduced to increase LS; in scenarios where the signal acquisition quality is poor, the weight ratio can be increased to prioritize imaging stability. On the other hand, based on a large amount of human data, the optimal weight ratio can be identified and used as a fixed parameter to achieve the optimal balance between signal anti-noise performance and image interpretability in most commonly used scenarios.

### 4.3. Advantages and Limitations of the Mixed Stimulation Pattern

The opposite stimulation pattern and adjacent stimulation pattern each have unique advantages and disadvantages in pulmonary EIT imaging: the opposite stimulation has strong anti-noise performance, but the overlapping of left and right lung ventilation images leads to difficulties in image interpretation; the adjacent stimulation yields pulmonary ventilation images with better interpretability, yet it is highly susceptible to noise and its imaging stability is inferior to that of the opposite stimulation. Therefore, in practical applications, EIT researchers and practitioners often face a dilemma of choosing between anti-noise performance and image interpretability. To address this dilemma, previous studies on pulmonary EIT stimulation patterns have proposed different optimization approaches. Yang et al. employed machine learning methods to generate stimulation patterns with the goal of optimizing EIT imaging quality, achieving certain improvements in specific scenarios [38]. However, the stimulation pattern is not the sole factor influencing imaging quality, and it is challenging to eliminate the impact of other variables (such as the types and parameters of regularization) during the optimization process. Consequently, these approaches face limitations in practical applications. Another approach, the rotating radius stimulation pattern [39], offers novel potential but lacks theoretical grounding, making standardization difficult. By contrast, the mixed stimulation pattern provides an excellent solution, which, to a considerable extent, integrates the advantages of the two traditional stimulation patterns and achieves mutual complementarity. It strikes a good balance in terms of the number of stimulation and measurement channels, EIT signal anti-noise performance, image interpretability, and stability in low-current scenarios. Consequently, the mixed stimulation pattern possesses the following advantages: (1) balanced performance; (2) high practicality; and (3) solid practical application foundation and standardization.

**Figure 10:**
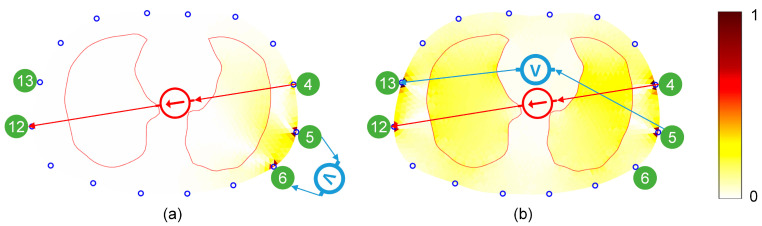
Sensitivity distribution across different measurement patterns under the same stimulation pattern. The number indicates the electrode number, and the arrow indicates the direction of current injection or voltage measurement. (**a**) opposite stimulation/adjacent measurement; (**b**) opposite stimulation/opposite measurement.

However, the mixed stimulation pattern is still limited to the Skip-*n* pattern, and its anti-noise performance, as well as various image indicators, remain between those of the two traditional patterns. The Skip-*n* pattern performs measurements via adjacent electrodes. According to the reciprocity theorem, the measurement results are consistent when the ports of the current source and the voltmeter are swapped. Therefore, the opposite stimulation with the adjacent measurement pattern is actually equivalent to the adjacent stimulation with the opposite measurement pattern, which does not break out of the framework of adjacent stimulation. EIT stimulation patterns in other application scenarios (prostate cancer detection [40] and stroke lesion detection [41,42]) provide new insights for optimizing pulmonary EIT stimulation patterns. These patterns optimize EIT imaging by constraining independent measurements, with the optimization objectives being maximum stimulation electrode distance or sensitivity within the region of interest. They also hold potential for pulmonary applications. These patterns feature opposite stimulation-opposite measurement configurations. Using a simplified 2D chest FEM, we calculated sensitivity distributions for opposite stimulation/adjacent measurement and opposite stimulation/opposite measurement, deriving lung region sensitivity (Figure 10). Results demonstrate a 10.5-fold (3.58:0.34) advantage in lung region sensitivity for opposite stimulation-opposite measurement configurations. Therefore, the mixed stimulation pattern has the following weaknesses: (1) it has not broken free from the traditional framework, and (2) it does not possess optimal performance.

In further research, we can start with the measurement pattern and explore stimulation–measurement patterns that are more suitable for pulmonary EIT imaging. In addition, LS cannot fully reflect imaging accuracy. Hence, it is necessary to explore evaluation indicators that can more accurately reflect imaging accuracy by combining the characteristics of EIT imaging and lung ventilation, and further optimize the stimulation and measurement patterns based on these indicators.

## 5. Conclusions

Aiming at the dilemma faced by EIT researchers and practitioners of having to choose between anti-noise performance and image interpretability when determining the stimulation pattern, this study proposed a novel mixed stimulation pattern, which has the following advantages: (1) The number of SMCs is 200, falling between that of the traditional opposite stimulation (192) and adjacent stimulation (208) patterns, which meets the real-time requirements during pulmonary ventilation monitoring. (2) It achieves a balance between the anti-noise performance of EIT signals and image interpretability, i.e., while ensuring SNR_p_, it enhances LS (and avoids excessive LS at the same time) and reduces RA and BA. (3) It provides a more flexible trade-off parameter *w*_op_:*w*_ad_, improving the adaptability of EIT to acquisition environments. (4) It still maintains stable imaging quality in low-current scenarios, i.e., a stimulation current amplitude of 600 μA can ensure the same imaging quality as 1 mA, and a stimulation current amplitude of 200 μA ensures the stability of most image and clinical indicators except for GI and RVD_SD_. Therefore, it provides a better choice of stimulation and measurement pattern for pulmonary EIT imaging.

## Figures and Tables

**Figure A1:**
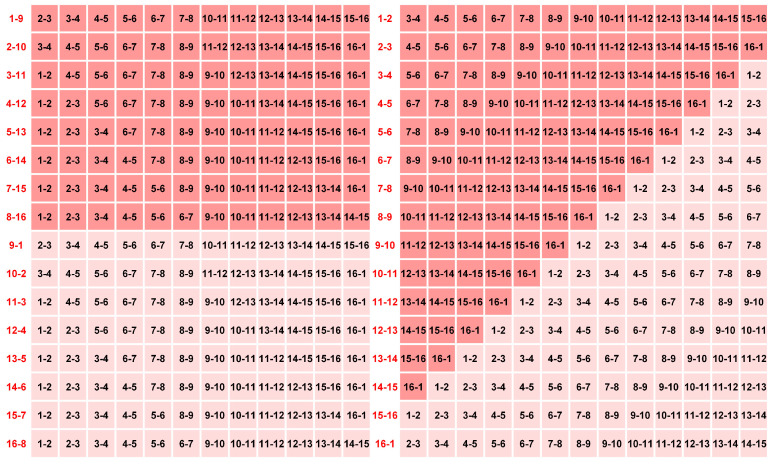
Stimulation and measurement channels included in the mixed stimulation pattern. The left and right parts represent all channels of the opposite and adjacent stimulation patterns, respectively. Among them, the white blocks with red fonts are stimulation channels, and the corresponding red blocks with black fonts are measurement channels. The dark red blocks are the measurement channels of the mixed stimulation pattern, while the light red blocks are the removed redundant measurement channels.

## Data Availability

Data are available upon reasonable request to the corresponding author.

## Appendix A

We provide the stimulation and measurement channels of the mixed stimulation pattern for the 16-electrode EIT system, as shown in Figure A1.
